# Exposure of Larvae to Sublethal Thiacloprid Delays Bee Development and Affects Transcriptional Responses of Newly Emerged Honey Bees

**DOI:** 10.3389/finsc.2022.844957

**Published:** 2022-04-05

**Authors:** Bin Li, Li Ke, Ai-Rui Li, Qing-Yun Diao, Qiang Wang, Yong-Jun Liu

**Affiliations:** Department of Honeybee Protection and Biosafety, Institute of Apicultural Research, Chinese Academy of Agricultural Sciences, Beijing, China

**Keywords:** honeybee (*Apis mellifera* L.), larva, thiacloprid, development, transcriptome (RNA-seq)

## Abstract

Understanding the cause of honey bee (*Apis mellifera*) population decline has attracted immense attention worldwide in recent years. Exposure to neonicotinoid pesticides is considered one of the most probable factors due to the physiological and behavioral damage they cause to honey bees. However, the influence of thiacloprid, a relatively less toxic cyanogen-substituted form of neonicotinoid, on honey bee (*Apis mellifera L*.) development is not well studied. The toxicity of sublethal thiacloprid to larvae, pupae, and emerging honey bees was assessed under laboratory conditions. We found that thiacloprid reduced the survival rate of larvae and pupae, and delayed the development of bees which led to lower bodyweight and size. Furthermore, we identified differentially expressed genes involved in metabolism and immunity though RNA-sequencing of newly-emerged adult bees. GO enrichment analysis identified genes involved in metabolism, catalytic activity, and transporter activity. KEGG pathway analysis indicated that thiacloprid induced up-regulation of genes related to glutathione metabolism and Toll-like receptor signaling pathway. Overall, our results suggest that chronic sublethal thiacloprid can affect honey bee colonies by reducing survival and delaying bee development.

## Introduction

Pollinating insects play a crucial role in maintaining a balanced ecosystem and agricultural production (1). Honey bees, one of the most important groups of pollinating insects, play an irreplaceable role in the pollination of crops and flowering plants (2). However, in recent years, many studies have shown that honey bee populations are declining globally due to climate change (3), widespread use of agricultural pesticides (4, 5), intensive agricultural development (6, 7), habitat conversion (8, 9) and specific parasites (10, 11) such as bacteria and viruses. The alarming decline in honey bee population has attracted global attention.

One major reasons for the decline in honey bee populations is due to the increased use of neonicotinoid pesticides (12, 13), a broad-spectrum insecticide (14, 15), due to their low toxicity and high efficiency (16). The systemic nature of neonicotinoid insecticides allows it could be transported to the whole plant, including the roots, stems, leaves, nectar and pollen (17). Honey bees can get exposed to these insecticides through their nectar collecting behavior (18, 19), increasing the likelihood of colony contamination. One study reported that at least one neonicotinoid was detected in 75% of honey samples worldwide (20, 21), neonicotinoid was also detected in bee pollen (22) and even in fruits and leaves (23), suggesting that bee colonies may have been contaminated with neonicotinoid pesticides.

Neonicotinoid pesticides have been shown to reduce bumble bee productions and delay weight gain (24); causes deleterious effects on honey bee behavior and function, including impairment of smell and taste (25), foraging and homing ability (26–28), colony communication (25, 28–32), immune function (33), and memory (34). Neonicotinoids can also affect the fertility of male bees (35) and impair the bee queens reproductive function (36). These behavioral changes usually result in colony failure. In 2013, the EU imposed partial restrictions on three of the most widely used neonicotinoids (37). It was not until December 2018 that the EU completely banned the outdoor use of imidacloprid, clothianidin, and thiamethoxam (38), furthermore, thiacloprid has also been banned recently by the EU (39).

Thiacloprid, a cyanogen-substituted neonicotinoid compound, exhibited lower toxicity than nitro-substituted neonicotinoid like imidacloprid, clothianidin and thiamethoxam (40, 41). Recent research identified a single cytochrome P450 CYP9Q3, that metabolizes thiacloprid with high efficiency than imidacloprid (42). However, thiacloprid has been reported to affect honey bees behavior (43), immunity (44), colony fitness and reproduction under field conditions (45). Exposure of thiacloprid at ambient concentrations has been shown to cause transcriptional changes in mitochondrial related genes (46), whereas sub-lethal concentrations of thiacloprid negatively affect the foraging and mortality of solitary bees and delay larval development (47), although the negative effect of thiacloprid is mitigated by providing high nutritional food supplement (48, 49). Our recent study shows that sublethal dose of thiacloprid exposure in adult bees can reduce survival rates (48, 49) and perturb the gut microbiota (50). However, there are only a few studies on the sublethal effects of thiacloprid on the growth and development of honey bees.

In this study, we investigated changes in the survival and growth of honey bees that were exposed to thiacloprid at the larval stage (see “Experimental design”). In order to find out the transcripts of genes related to the development of honey bees, we performed abdominal transcriptome sequencing on the first day of adult honey bee emergence. Our results show that exposure to thiacloprid at the larva stage retarded larval growth and reduced survival rate perhaps by reducing energy supply through the downregulation of metabolism-related genes, such as amino acid metabolism, lipid metabolism, and sugar metabolism.

## Materials and Methods

### Chemicals and Solutions

Thiacloprid dry powder (catalog number 37905-100 mg-R, Sigma-Aldrich, St. Louis, MO, USA) 10 mg was dissolved in 100 ul dimethyl sulfoxide (DMSO) and then diluted into a thiacloprid stock solution (1,000 mg/L) by adding 9.9 mL sterile sucrose solution. The thiacloprid stock solution was aliquoted and stored in a−80°C freezer. During the experiment, the stock solution was thawed before each use and mixed with fresh royal jelly to the desired concentration (0.5, 1.0 mg/L) and fed to honey bee larvae. The control group of honey bee larvae were fed a fresh royal jelly diet without thiacloprid. Previous studies have shown that 0.1% DMSO had no effect on the feeding behavior of honey bees (51, 52), and the administration of food containing DMSO to honey bee larvae did not reveal any negative effects on the survival status of the bees (53). Our highest thiacloprid exposure concentration which contains 0.001% DMSO, the negative effect of DMSO in such a low concentration is negligible, therefore, we did not set a solvent-control group for further testing.

### Bees and Treatments

Honey bee (*Apis mellifera*) colonies were maintained at the Institute of Apicultural Research apiary (40°00043" N, 112°12043" E), Chinese Academy of Agricultural Sciences (Beijing, China). Instar larvae were randomly collected from four healthy colonies without history of pesticide exposure or other bee diseases. The honey bee colonies were strictly monitored to check their health status. Larvae samples were separated into eight replicates for each treatment condition. Each replicate contains 48 larvae in total. The honey bee queens were placed on an empty comb kept in a queen excluder push-in cage. 24 h later, the queens were released, and the combs were left in the hives for 3 days during the egg stage until the larvae hatched. On the 4^th^ day early morning, the combs were transported into the laboratory and the newly hatched worker bee larvae were transferred into sterilized polystyrene grafting cells of a 48-well culture plate. The plates were incubated in an artificial climate box (RXZ - 380C, Ningbo, China), with a relative humidity of 90 ± 5% and temperature of 35°C, under dark conditions. The larvae food contains royal jelly, glucose, fructose, yeast extract, and water. They are mixed in certain proportions and feed based on different development stages. Details of honey bee larvae rearing protocols referenced are described in Daniel R Schmehl (54). The development of the honey bees has 3 stages. From the 4^th^ day to the 10^th^ day is larval stage, from the 11 to 20^th^ day is pupal stage, and after the 21^st^ day is adult stage (i.e., the bees emerge from the hive).

To study the effects of thiacloprid exposure on honey bee development, two different larvae exposure concentrations of thiacloprid were used, 0.5 mg/L (T0.5) and 1.0 mg/L (T1.0). The concentration gradient was set up in an attempt to recreate the true concentration detected in the hive in the wild. T0.5 and T1.0 represented the average (33, 47) concentration of thiacloprid detected in the environment. Royal jelly diets containing 0 mg/L (T0), 0.5 mg/L (T0.5), and 1.0 mg/L (T1.0) concentrations of thiacloprid were fed to honey bees during the entire larva stage, which is the 4th day to the10th after the queen laid eggs. During the pupa stage around day 10, and all the alive pupae were transferred into 24-well plate with a piece of Kimwipe lining the bottom of each well, and the humidity inside the incubator was dropped to 75 ± 5% during the pupa stage from the 11 to the 20^th^ day.

The number of pupae formed were counted on the 10^th^ day and the pupa rate was calculated as the ratio of live pupa to the number of transferred larvae. The weight of pupation was recorded on the 1^st^ day of pupa stage and on the 10^th^ day. On the 21^st^ day, the adult bees emerged and the emergence rate of bees was calculated as the ratio of the total number of emerged bees to the total number of transferred larvae on the 10^th^ day. The number of emerged bees was recorded at 8 AM in the morning and 3 PM in the afternoon on the 21^st^ day. Furthermore, the newly emerged adult bee weight was recorded. The survival rate of bees was calculated according to the number of dead and alive bees for the whole development period.

### Honey Bee Larvae Growth and Development: Size Assessment

From larva stage to pupa stage, we took photos of individual bees each day to assess their growth and development with a high-resolution camera (Dino-Lite AM4815 Series, Taiwan, China). Then, the images were exported to Image J (National Institute of Mental Health, USA) for size assessment analysis. The borders along the whole body was plotted and converted to grayscale, and the area sizes of the whole body was measured.

### RNA Extraction and RNA Sequencing Analysis

On the first day the bee larvae emerged, 5 bees from each treatment groups (T0, T0.5 and T1.0) were randomly collected, and each bee from the same treatment group was considered as one RNA replicated sample. A total of 15 bees were used to measure the honey bee abdominal transcriptome. The whole abdomen tract of bees were carefully separated and transferred to 1.5 ml fresh centrifuge tube with sterile forceps respectively. The centrifuge tube storing the abdominal tract of bees were frozen and stored at−80°C until the RNA is extracted for further use. RNA from the honey bee abdominal tract was extracted following the protocol provided by the TRIzol manufacturer. The concentration and purity of the extracted RNA was measured using a Nano Drop 2000 spectrophotometer, and the RNA integrity number was measured using 2100 Bioanalyzer (Agilent Technologies, California, USA). RNA sequencing libraries of qualified sample RNA (OD260/280 =1.8~2.2, OD260/230 ≥ 2.0, RIN ≥ 6.5, 28S:18S ≥ 1.0, >1μg) was used to construct the sequencing library using Illumina TruseqTM RNA sample preparation Kit (San Diego, CA). High-throughput sequencing was performed on Illumina Novaseq 6000 sequencing platform (Shanghai, China) provided by Shanghai Majorbio Bio-Pharm Technology Co., Ltd. After sequencing, the raw reads were filtered for low quality sequences and adapter sequences were cropped using SeqPrep. Sequence reads with Q-score <30 and length <50 bp were removed from the dataset, and the remaining sequences were used for bioinformation analysis.

The clean reads were obtained through quality control, and a total of 12,373 expressed genes were identified by mapped to the reference genome (https://www.ncbi.nlm.nih.gov/genome/?term=apis mellifera). The remaining genes that cannot be annotated with known genes may correspond to non-coding regions, non-coding RNAs, or short sequences that do not contain known protein structural domains.

The gene expression levels were measured using fragments per kilobases per million reads (FPKM). Data analysis was performed by using the Majorbio I-Sanger online cloud platform (www. i-sanger.com). The differentially expressed genes (DEGs) between the control (T0) and treatment (T0.5, T1.0) groups was identified by DEGseq2 software based on negative binomial distribution. The significance of DEGs was determined based on the Benjamini and Hochberg's method adjusted *P*-value <0.05 & |log2(Foldchange)| ≥1. The functional classification of DEGs and functional enrichment analysis were realized out using Gene Ontology (GO) (http://www.geneontology.org) (55) and Kyoto Encyclopedia of Genes and Genomes (KEGG) databases [http://www.genome.jp/kegg/ (56)].

### Statistical Analyses

Statistical analyses for the three different concentrations of thiacloprid on honeybee survival rates were performed using the log-rank test. Wilcoxon rank-sum test was used to compare pupation rate, new emergence rate and gene expression between treatment groups (T0.5 and T1.0) and control group (T0) and the original *P*-values were corrected and visualized using the Bonferroni procedure for multiple comparisons. Analyses were executed using GraphPad Prism (GraphPad Prism Software 9.1.2, San Diego, CA, USA). The significance of *P* < 0.05 was considered statistically significant.

## Results

### Thiacloprid Affects Honey Bee Survival and Reduces Pupation and Emergence Rates

**Figure 1:**
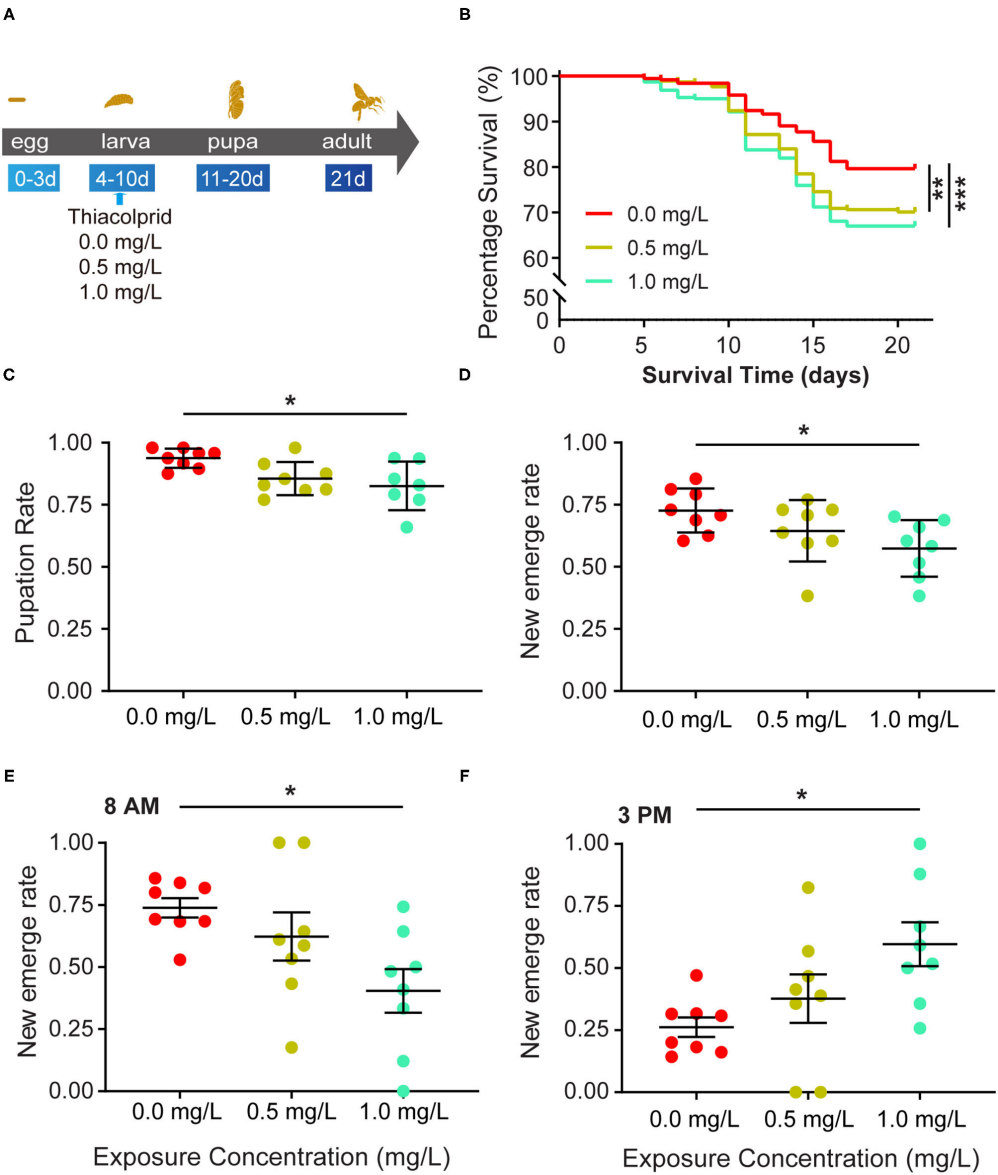
Effects of thiacloprid on the survival, pupation and emergence status of honey bee (*Apis mellifera*) larvae. **(A)** Schematic view of the experiment. Numbers indicate day of development after egg laying. Honey bee larvae were exposed to different concentrations of thiacloprid in larva stage from the 4^th^ day to the10^th^ day and incubated for 11 days until emergence as adult bees. During the rearing period, honey bee conditions were recorded daily to evaluate honey bee growth and development. In addition, samples were collected at 21 days in order to study the molecular effects of thiacloprid on bees. **(B)** The effect of three different concentrations of thiacloprid on the survival rate of honey bee larvae over time. Survival curves are plotted with different colors per rearing context, red for non-thiacloprid exposure group, yellow is for 0.5 mg/L treatment group, and green is for 1.0 mg/L treatment group. Log-rank (Mantel-Cox) test (**p < 0.01, ***p < 0.001) was used for statistical analysis. **(C)** Pupation rate of honey bee larvae in the three different thiacloprid treatments. The pupation rate decreased in a dose-dependent manner when larvae are fed with thiacloprid. **(D)** Honey bee emergence rate in three different concentrations of thiacloprid treatments. **(E,F)** The percentage of emergence of adult honey bees were counted at 8 AM **(E)** and 3 PM **(F)**. Error bar is SEM. Wilcoxon rank sum test, adjusted for multiple comparisons, *p < 0.05 when compared to the respective controls.

Honey bees were exposed to three different concentrations (0, 0.5, and 1.0 mg/L) of thiacloprid during the larva stage from the 4^th^ to the 10^th^ day as illustrated in Figure 1A. From the 5 to the 21^st^ day, honey bee survival rate was calculated on each day. Figure 1B shows the survival rate in the three groups (T0, T0.5, T1.0) (Figure 1B). The decrease in the survival rate of honey bee larvae in the control group represents the survival rate of honey bee larvae in a natural state without the influence of thiacloprid. Analysis of survival rate revealed that both low (T0.5) and high (T1.0) concentrations of thiacloprid led to a significant decline in survival rate of honey bees compared to the control group (T0) (Log-rank test, T0.5 vs. T0, *p* = 0.002; T1.0 vs. T0, *p* < 0.0001). However, there is no significant difference in the survival rate of honey bees between T0.5 treatment group and T1.0 treatment group (Log-rank test, *p* = 0.334). During the entire growth and development period of the honey bee larvae, honey bee survival decreased by 9 and 12% in the low concentration group (T0.5) and high concentration group (T1.0) when compared to the control group (T0). The pupae of honey bee larvae formed around the 10^th^ day, the pupation rate of honey bee larvae in high (T1.0) concentration group decreased by 11% (T1.0 group vs. T0 control: 0.823 ± 0.036 vs. 0.938 ± 0.036), which is significantly lower than in control group (T0) in Figure 1C (Wilcoxon rank-sum test, *p* =0.021, adjusted for multiple comparisons). However, the pupation rate of honey bee larvae in the low concentration group (T0.5) was similar to the non-treated control group (T0) with an 8% decrease of pupation.

The honey bee pupae develop into adults and emerge around the 21^st^ day. We measured the honey bee adult emergence rate of each thiacloprid treatment groups (T0, T0.5 and T1.0). The thiacloprid treatments (T0.5 and T1.0) lead to substantially reduced emergence rates of adult honey bees compared to non-thiacloprid treated control group (T0). Although the rate of honey bee adult emergence in the low concentration group (T0.5) was similar to that of the control group (T0) (Wilcoxon rank-sum test, *p* = 0.206) (Figure 1D), adult honey bee emergence from the high concentration group (T1.0) was significantly lower than the control group (T0) (T0 vs. T0.5, *p* = 0.240; T0 vs. T1.0, *p* = 0.010, Wilcoxon rank-sum test, adjusted for multiple comparisons). Overall, new emergence rates are0.57 ± 0.043 for T1.0 group, and 0.727 ± 0.043 for control group. Our data suggests that the honey bee adult emergence rate decreases in a dose-dependent manner when larvae are treated with thiacloprid.

Furthermore, in order to assess whether the larva treated with thiacloprid will prolong honey bee adult emergence time, we calculated the adult emergence rate at 8 AM on the 20^th^ day and at 3 PM on the 21^st^ day. The result illustrates that honey bee adult emergence at 8 AM reached 72% in the control group (T0), 62% in the low concentration group (T0.5), and only 40% in the high concentration group (T1.0). There was a significant difference between the control (T0) and high concentration groups (T1.0) (Wilcoxon rank-sum test, *p* = 0.035) (Figure 1E). At 3 PM, the emergence rate of adult honey bee reached 28% in the control group (T0), 38% in the low concentration group (T0.5), and 60% in the high concentration group (T1.0). Again, there was a significant difference between the control (T0) and high concentration groups (T1.0) (Wilcoxon rank-sum test, *p* = 0.042) (Figure 1F).

### Thiacloprid Suppresses Honey Bee Larval Growth and Affects Newly Emerged Adult Size

**Figure 2:**
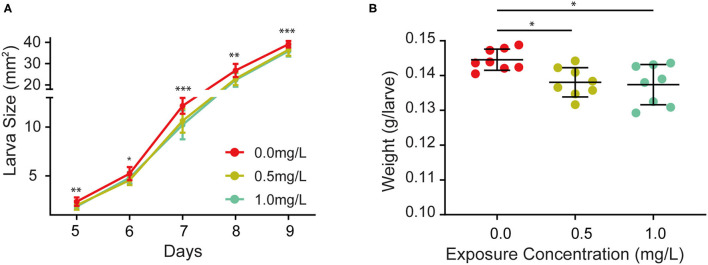
Effects of thiacloprid on growth and developmental size and weight of honey bees at larval stage. **(A)** Effect of thiacloprid on the growth and developmental size of honey bee larvae at the larva stage. ANOVA test, *P < 0.05, **p < 0.01, ***p < 0.001. **(B)** Weight of fully grown larvae for the three treatment groups. Values represent mean ± SEM. Wilcoxon rank sum test (*p < 0.05) was used for statistical analysis.

In order to investigate whether thiacloprid has any effects on larval growth, larva body size, a known variable to estimate growth development, was measured consecutively for 5 days during the larva stage in units of area (mm^2^) in Figure 2A. Examples of larvae from the control group (T0) and the two treatment (T0.5 and T1.0) groups are shown in Supplementary Figure 1. The size of larvae in the T0.5 and T1.0 treatment groups were smaller than that of the control group, whereas larvae sizes of the thiacloprid treated groups were similar. We find that during the 5 days of larval development in Figure 2A, larval growth changes exponentially in size. Compared to the control group, the honey bee larvae from the thiacloprid treated groups decreased in overall body size (Wilcoxon rank-sum test, ^*^p < 0.05, ^**^ *p* < 0.01, ^***^p < 0.001). However, this phenotype was not concentration-dependent and developmental delay effects are similar in both the low concentration group (T0.5) and the high concentration group (T1.0). Additionally, we measured the larval body weight during the transition from honey bee larva to the pupa on ~the 10^th^ day, and we found a marked reduction in larval body weight when the larvae were fed with a low (T0.5) or a high (T1.0) concentration of thiacloprid (Wilcoxon rank-sum test, *p* < 0.05) (Figure 2B). Altogether, our data shows that honey bee larvae consuming a diet with thiacloprid at concentrations of T0.5 and T1.0 induced growth defects.

**Figure 3:**
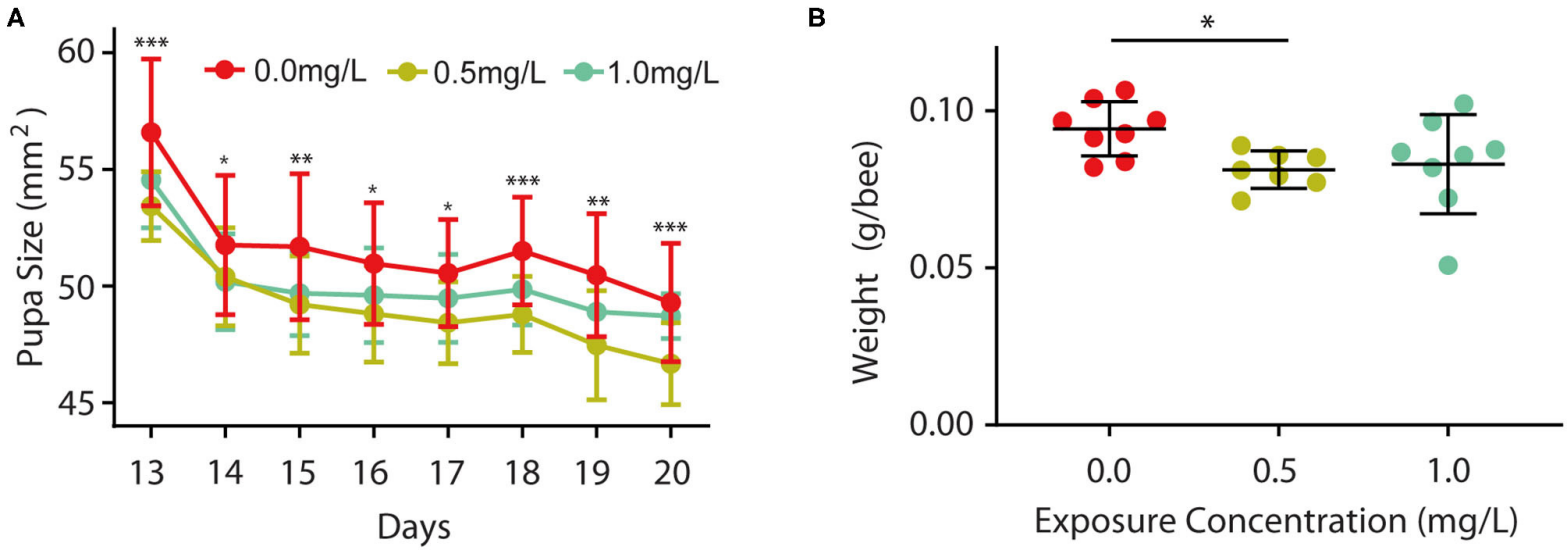
Effects of thiacloprid on growth and developmental size at pupal stage, and weight of honey bees after emergence. **(A)** Effect of thiacloprid on growth and developmental size of honey bee larvae in the pupal stage. Values represent mean± SEM. ANOVA test, *P < 0.05, **p < 0.01, ***p < 0.001, *P* < 0.05. **(B)** Effect of three different concentrations of thiacloprid treatments on the weight of emerged honey bees on the 20^th^ day. Wilcoxon rank sum test, *p < 0.05.

Furthermore, we wondered whether thiacloprid also affected body size during the pupa stage, so we examined the size of the honey bee pupae in the control and thiacloprid treated groups. The size of the pupae exposed to thiacloprid in the larvae stage was smaller than the size of pupae formed by control larvae (Supplementary Figure 2). Pupae body size was measured consecutively across the 8 days of pupal development shown in Figure 3A. When compared to the control group (T0), we observed a decrease in body size in both the low concentration group (T0.5) and the high concentration group (T1.0) (Wilcoxon rank-sum test, *p* < 0.05) (Figure 3A). Interestingly, both groups vary in size, but the bee pupae gradually become smaller as the days progress. On the 20^th^ day that the bee emerges, the weight of newly emerged adult worker honey bees from the lower thiacloprid-treated group (T0.5) was significantly different from that of the control group (T0) (Wilcoxon rank-sum test, *p* = 0.01); there was no significant difference in the gross weight of emerged bees between higher thiacloprid-treated group (T1.0) and control group (T0) (Figure 3B) (Wilcoxon rank-sum test, *p* = 0.09). In summary, larvae treated with thiacloprid showed significant delay in development compared to the control group when comparing body size and weight during both larva stage and pupa stage.

### Raw RNA Sequencing Data Analysis

We used high-throughput RNA-Seq to analyze the dose-dependent changes in transcripts under the different concentrations of thiacloprid. The RNA-seq analysis obtained about 761160730 clean reads and 112554833766 clean bases from 112.55 Gb of clean sequencing data of all biological repeats, with an average GC content of 42.06% and a sequencing error rate of <0.0256%. The Q30 of all 15 samples ranged from 93.66 to 94.06%, with an average of 93.87%. These clean reads were mapped to known reference genomic libraries and at least 93.16% successfully matched to single or multiple genomic positions from each library. The main sequencing assembly information is summarized in Supplementary Table 1. A total of 12,125 genes were identified by sequencing analysis. Principal component analysis (PCA) in Supplementary Figures 1, 3A showed that three groups were clustered in the thiacloprid-treated and non-treated control samples, while the 1.0 mg/L (T1.0) thiacloprid group differed the most from the control group (T0). These results demonstrate the reliability and stability of the RNA-seq results.

### Differentially Expressed Genes After Thiacloprid Exposure in Larva Stage

**Figure 4:**
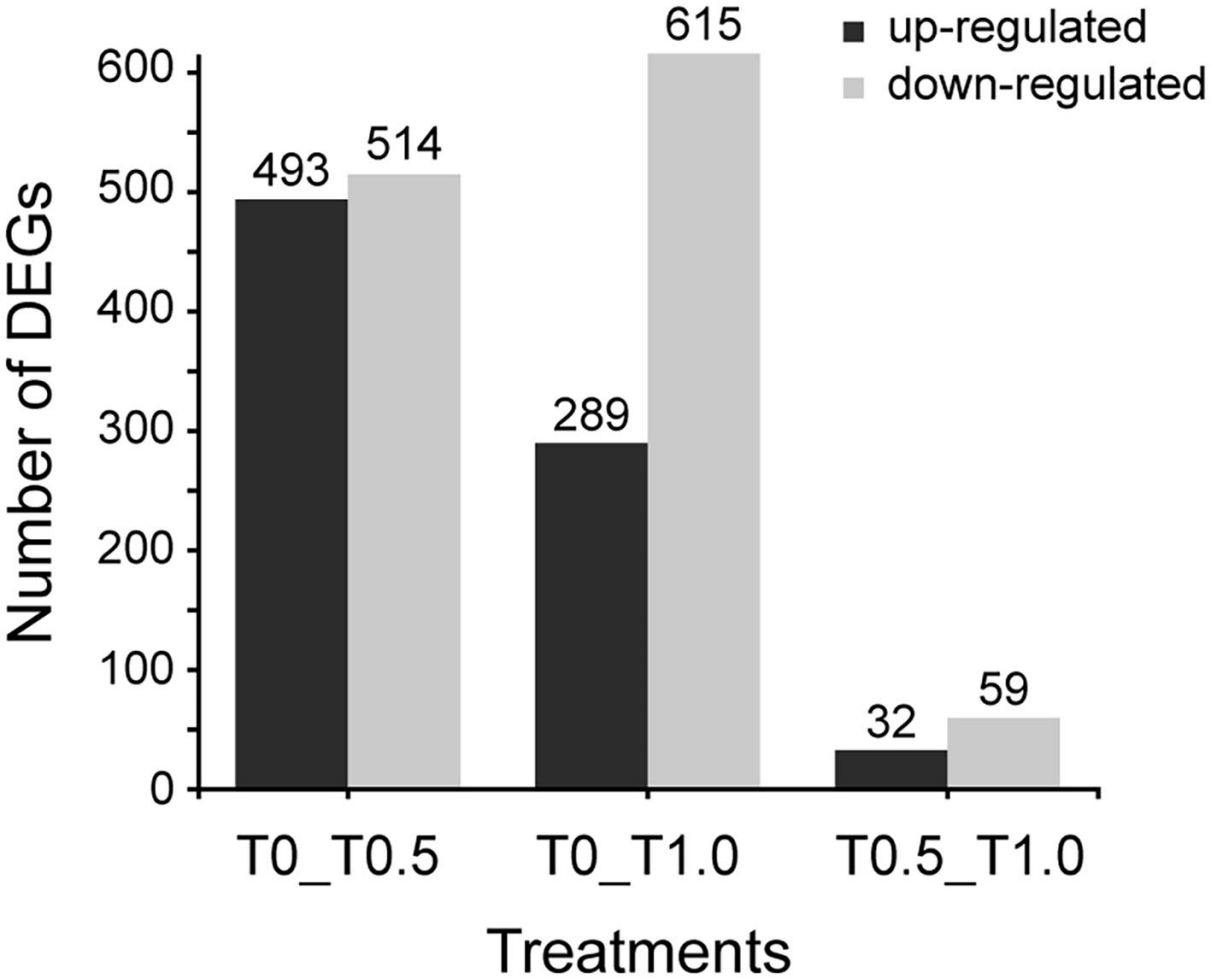
Numbers of differentially expressed genes (DEGs) of *A. mellifera* larvae treated with 0.5 mg/L and 1.0 mg/L thiacloprid. Up- and down-regulation indicates that these genes are expressed higher or lower in the treatment groups (T0.5 and T1.0) compared to the control (T0), or genes expressed higher or lower in the higher concentration group of T1.0.

When compared to the control group (T0), 1692 differential expressing genes (DEGs) were identified between two thiacloprid treated groups (T0.5 and T1.0). However, it seems like the effect is not related to thiacloprid exposure concentrations because low concentration (T0.5) lead to 1007 DEGs and high concentration (T1.0) lead to 904 DEGs. The number of up-regulated DEGs decreased while the number of down-regulated DEGs increased with exposure to increasing concentrations of thiacloprid (Figure 4). Of these DEGs, 493 genes were up-regulated and 514 genes were down-regulated in the 0.5 mg/L thiacloprid-treated group (T0.5) compared to the control group (T0). While there were 289 up-regulated genes and 615 down-regulated genes in the 1.0 mg/L thiacloprid-treated group (T1.0) compared to the control group T(0) (Figure 4, Supplementary Table 2). Furthermore, comparing the 0.5 mg/L thiacloprid treated group (T0.5) with 1.0 mg/L thiacloprid group (T1.0), 32 were up-regulated genes and 59 were down-regulated genes (Figure 4, Supplementary Table 2). By Venn analysis in Supplementary Figures 2, 3B, we found that 79 genes were upregulated and 140 genes were downregulated in the thiacloprid treated groups (T0.5 and T1.0) compared to the control group (T0).

### Functional Annotation and Classification of Differentially Expressed Genes

Compared to the control group, a total of 219 differentially expressed genes (DEGs) were identified from two thiacloprid-treated groups (T0.5 and T1.0). Among them, 181 genes were annotated with information, and 38 genes were annotated as “uncharacterized.”

The biological functions of the 219 differentially expressed genes (DEGs) were classified into 29 functional groups according to the GO database, which were mainly organized into three categories: molecular function, cellular component, and biological process (Supplementary Figure 3C, Supplementary Table 3). According to GO term classification, cellular component accounts for a majority of the GO category (38.0%), followed by biological processes (31.0%) and molecular function (31.0%). Among the categories, “binding” (GO: 0005488, 61 genes, 27.9%), “catalytic activity” (GO: 0003824, 57 genes, 26.0%), “membrane fraction” (GO: 0044425, 54 genes, 24.7%), “cell part” (GO: 0044464, 52 genes, 23.7%), “cellular process” (GO: 0009987,46genes, 21.0%), “metabolic process” (GO: 0008152,37genes, 16.9%), “protein-containing complex” (GO: 0032991,28genes, 12.8%), “organelle” (GO: 0043226,28genes, 12.8%), “organelle fraction” (GO: 0044422,18genes, 8.2%), and “bioregulation” (GO: 0065007,16genes, 7.3%) were the most abundant. Only one gene was enriched in some GO categories (GO: 0032501, GO: 0022610, GO: 0032502, GO: 0005623, GO: 0044421, GO: 0030054, GO: 0140104, GO0016209) because of the low number of some single genes.

**Figure 5:**
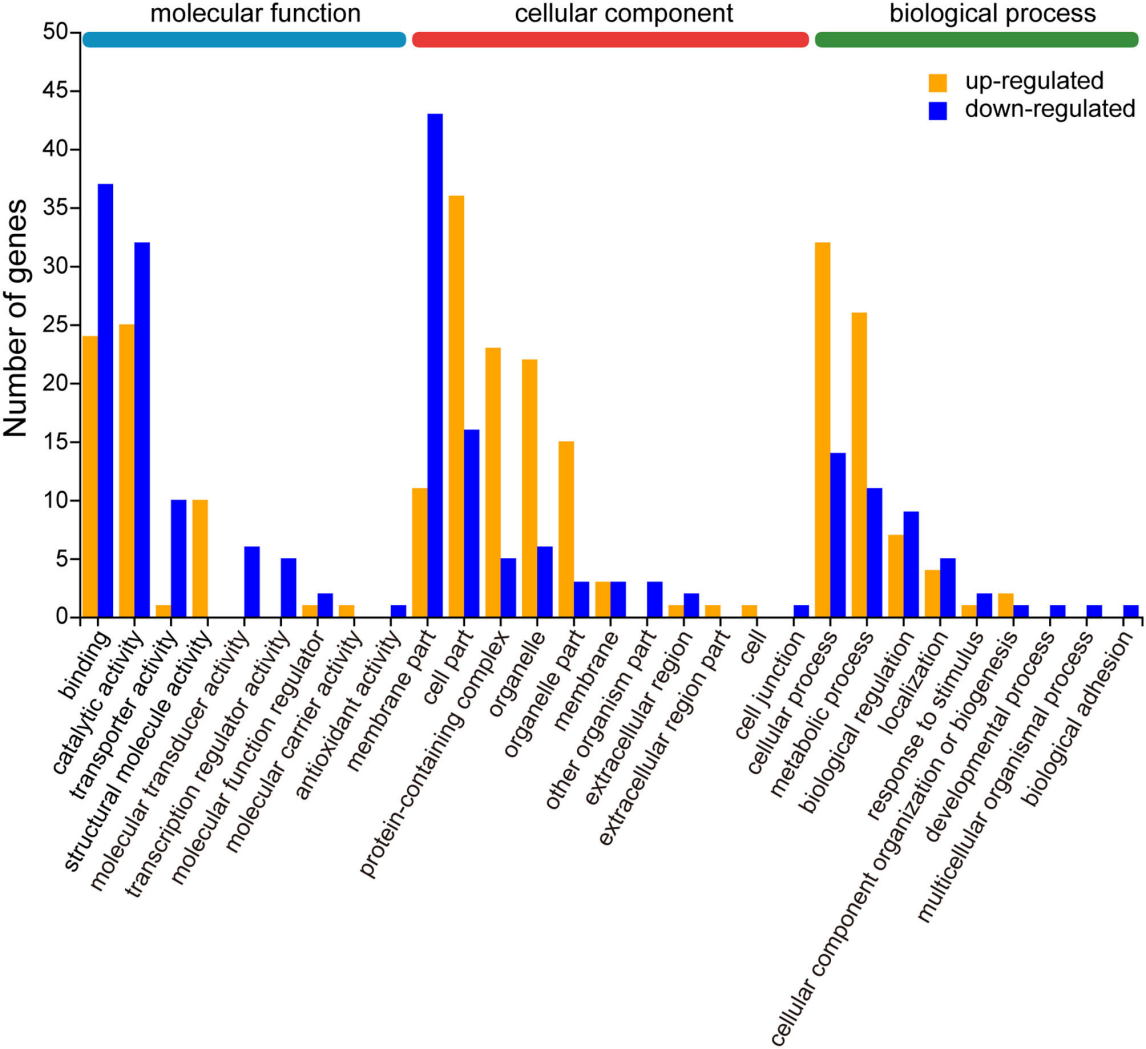
Gene ontology (GO) enrichment analysis of differentially expressed genes (DEGs) for up- and down-regulation patterns. The results are located in three main categories: molecular function, cellular component, and biological process. The upward mode contains 21 GO terms and the downward mode contains 25 GO terms. The yellow and blue bars represent the DEGs from the up- and down-regulation, respectively. The X-axis indicates the second category of GO terms and the Y-axis indicates the number of DEGs.

To further evaluate the effectiveness of the annotation process, we visualized 21 up-regulated and 25 down-regulated DEGs by GO enrichment analysis (*p* < 0.05). In most GO terms, the number of down-regulated DEGs was more than the number of up-regulated DEGs, such as “binding,” “catalytic activity,” and “transporter activity” in the molecular function category, and “membrane part” and “extracellular region” in the cellular component category. In contrast, the number of up-regulated DEGs was more than the number of down-regulated DEGs were enriched in “cell part,” “protein-containing complex,” “organelle,” and “organelle part” in molecular function category, and also in “cellular process” and “metabolic process” in biological process category. Other than that, only a few functional categories are individually up-regulated or down-regulated (Figure 5, Supplementary Table 4).

### KEGG Pathway Analysis

**Figure 6:**
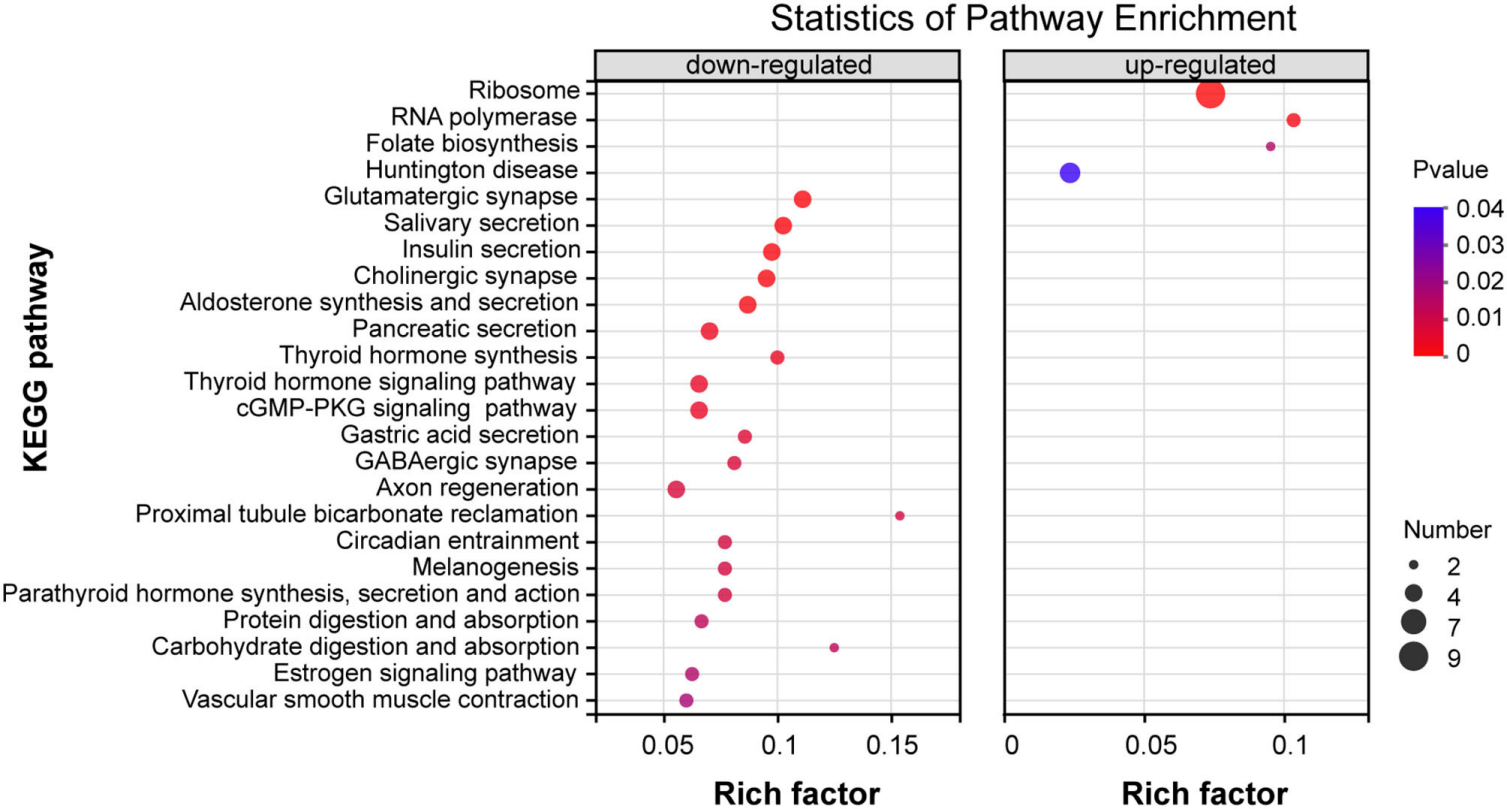
KEGG enrichment pathway analysis of differentially expressed genes (DEGs) for down-regulation patterns (left panel) and up-regulation patterns (right panel) . The y-axis indicates pathway names, the x-axis indicates rich factors corresponding to the pathways, *p*-value is represented by the color of the dots, and the number of DEGs expressed in each pathway is represented by the size of the dots.

To address the potential pathways of DEGs, we characterized these 219 DEGs of the thiacloprid treated groups and non-exposure control group using KEGG annotation and related pathways. The top 20 KEGG enriched pathways DEGs up- and down-regulated clusters are presented in Figure 6, Supplementary Table 5. Among the DEGs within the down-regulated clusters the enrichment pathways are mainly related to metabolism, organismal systems, cellular processes, and environmental information processing, such as glutamatergic synapses, salivary secretion, insulin secretion, cholinergic synapses, aldosterone synthesis and secretion, pancreatic secretion, thyroid hormone synthesis, thyroid hormone signaling pathway, and cGMP-PKG signaling pathway, which were significantly enriched (*P* < 0.05). Though not reaching statistical significance, some other pathways are also mainly related to metabolism including: organismal systems and environmental information processing, such as fat digestion and absorption, glycolysis/gluconeogenesis, cAMP signaling pathway and MAPK signaling pathway-fly (Supplementary Table 5). DEGs within the up-regulated clusters (Figure 6), enriched pathways include ribosomes, RNA polymerase, folate biosynthesis, all of which were significant (*p* < 0.05). Pathways that are not significantly different, such as cysteine and methionine metabolism, riboflavin metabolism, sulfur relay system, pantothenic acid and CoA biosynthesis, and fatty acid biosynthesis are also included in Supplementary Table 5.

Our results showed that thiacloprid treatments induced larvae developmental delay, which might be caused by metabolic dysfunction. Therefore, we further analyze the metabolism-related pathways. A total of 12 DEGs were assigned to metabolism-related pathways, including amino acid metabolism, lipid metabolism, carbohydrate metabolism, and vitamin metabolism. Among them there are 16 down-regulated metabolic pathways and 15 up-regulated metabolic pathways (Supplementary Table 6-Sheet1). The down-regulated metabolic pathways are mainly related to fat and carbohydrate metabolism, which is consistent with the decrease in body size and weight found in the thiacloprid treated groups during the larva stage.

With further analysis, a total of nine immune pathways were identified (Supplementary Table 6-Sheet2), including Toll-like receptor signaling pathway, NOD-like receptor signaling pathway, Fc gamma R-mediated phagocytosis, Fc epsilon RI signaling pathway, B-cell receptor signaling pathway, and leukocyte transendothelial migration and chemokine signaling pathway, natural killer cell-mediated cytotoxicity, and platelet activation. We also focused on some pathways related to signal transduction, which have a total of 21 (Supplementary Table 6-Sheet3) enriched pathways. Some of which are also related to immune regulation, such as VEGF, Ras, PI3K-Akt, MAPK, Rap1, cAMP, phosphatidylinositol signaling pathway. Moreover, some pathways related to circadian entrainment, olfactory transduction, and aging are downregulated suggesting that the normality of honey bees has been adversely affected.

## Discussion

Neonicotinoids are known to have adverse effects on the health and performance of bees (24–28, 34, 43), yet there are barely any studies on the sublethal effects of relatively less toxic neonicotinoids, such as thiacloprid, on the growth and development of honey bees. In this study, we investigated the effects of exposure to three different concentrations of thiacloprid (0, 0.5 and 1.0 mg/L) on honey bee larval survival rate, larval weight, pupation rate, emergence rate, and overall developmental size. In addition, we studied the expression of honey bee transcriptome genes of newly emerged adult worker bees to further illustrate the effects of thiacloprid on developmental genes.

### Thiacloprid Affects Honey Bee Survival, Reduces Pupation and Emergence Rates

Young adult bees are vulnerable to contaminated pollen and nectar in the hive, making it likely that bees of all ages and castes have been exposed to pesticides (57). We found that honey bee larvae that were exposed to thiacloprid (T0.5, T1.0) had reduced the survival rate, pupation rate, and emergence rate compared to the control group, which indicates that pesticide exposure significantly reduces the survival of honey bee larvae (in Figure 1B) Moreover, our previous studies showed chronic exposure to thiacloprid decreased the overall survival rate of adult bees (50) and may reduce colony size (58) Although bee colonies are not negatively affected by chronic exposure to sublethal concentrations of thiacloprid under field conditions (38), this differential impact may be due to differences between the two studies, such as experimental conditions (e.g., field and laboratory), food sources, and colony status. In addition, thiacloprid is a neuro-affective toxin that affects the learning, memory, and homing ability of honey bees (43, 59). Further, thiacloprid can also affect the health of bees and reduce survival rates by affecting the blood cells and cystic membrane of honey bees (33).

During the honey bee pupation stage, the larvae will complete morphological developmental changes. Similar to the Tavares et al. (60) study, our findings showed that the pupation rate decreased with increasing concentrations of pesticide exposure because some of the larvae did not successfully turn into pupae, and died (Figure 1C). Moreover, the impact of thiacloprid on honey bee development is demonstrated through the significant reduction in the hive emergence rate (Figures 1D–F) and significant delay in the time of adult honeybee emergence, which is consistent with previous studies (61). The transitional period during metamorphosis is important for the developmental success of honey bees, and our data demonstrates that these stages are particularly susceptible to disruption after exposure to insecticides (60).

### Thiacloprid Retards the Developmental Size of Honey Bee Larvae

Differences in larval developmental size were observed in early larval stages (i.e., during larval feeding) since the second day of feeding on thiacloprid-containing food. On each day of this stage in Figures 2A, 3A (Supplementary Figures 1, 2), we found persistent differences in larval developmental size as well as significant differences in larval body weight. These findings are consistent with previous studies examining the effects of pesticide exposure on larval morphological size and body weight (62). This is most likely due to the fact that larvae are more sensitive to thiacloprid pesticides than adult bees. Furthermore, exposure of larvae to neonicotinoid pesticides continues to negatively affect their physiological condition as they transition into the pupal stage (63). After the adult bees emerge, the body weight of the bees in Figure 3B remained significantly lower, indicating that the negative effects of thiacloprid on the growth and development of bees were persistent throughout its life cycle.

### Effects of Thiacloprid Exposure on the Immune System of Honey Bees

Honey bees are social insects and their immunity patterns include individual immunity and herd immunity (64). In contrast to Drosophila melanogaster and mosquitoes, individual immunity genes of bees have been partly lost during the evolutionary process (65), which renders the honey bees more susceptible when exposed to the pesticides. There are approximately one-third of the 17 immune gene families in insects and is enriched in four related natural immune signaling pathways: Toll, Imd, Jak/STAT, and JNK (66). Understanding how these immune pathways play an important role in the regulation of immune homeostasis through negative feedback mechanisms facilitates the study of the effects of drug stress on honey bees' normal immune and growth functions.

In our study, a total of 9 pathways related to the immune system were annotated in Figure 5, one of which, the Toll pathway, was found to be upregulated under thiacloprid exposure, which is consistent with the immune response and activation of the Toll pathway when honey bees are exposed to aphidicolin and defensin (67). The KEGG analysis (Figure 6) showed that these differential genes were not only enriched in the immune system, but it is also associated with immune signaling pathways, including phototransduction fly pathway, VEGF, B cell, Ras, PI3K-Akt, MAPK, Rap1, cAMP, phosphatidylinositol signaling, and phosphatidylinositol metabolism (68). The immune genes of honey bees were activated under the stress of thiacloprid in the absence of biological effects induced by external factors, such as antimicrobials, aphidicolin, defensins and other insects. Differential changes in immune gene expression may lead to disorders and consequently have detrimental effects on honey bee health. The other part of the insect immune response occurs in the hemolymph system through a pre-phenol oxidase cascade reaction (69). The downregulation of platelet coagulation found in our study may indicate impairment of coagulation and phenol oxidase cascade reactions, which could negatively affect the immune response of honey bees.

### Effects of Thiacloprid Exposure on Energy Metabolism and Physiological and Biochemical Responses of Honey Bees

Our study found a dose dependent effect of thiacloprid exposure leading to transcriptional alterations (mainly down-regulation) of genes in the abdomen of honey bees. Although different concentrations of thiacloprid feeding lead to various differential gene expression, some functional annotations and enrichment pathways are common. Significant differences in gene expression within common pathways were mainly related to metabolism, signal transduction and organismal systems (physiological and biochemical pathways) (Figure 5).

Opsins are an important component of the development of light-signaling receptors in the honey bee pupal stage and play an important role in regulating biological rhythms (70). We found significant down-regulation of pathways associated with circadian rhythms and phototransduction, which were most likely negatively affected by thiacloprid. In addition, we also found a down-regulation of Ac3, a gene related to olfaction, suggesting that thiacloprid may have influenced the odor judgment of honey bees (68). The visual and olfactory systems play an important role in insect mating, nursing, gathering, defense, feeding and “social” interactions (71, 72). The differentially expressed genes caused by thiacloprid exposure may affect the reproductive and foraging behavior of honey bees, which in turn may negatively affect the reproduction and development of bee colonies.

Differential changes in genes associated with metabolism are likely to adversely affect the physiology and performance of honey bees, including survival status, foraging time, communication, and flight outings (44, 73). It may also affect the transition of nurse bees to foraging bees (73, 74). In addition, metabolic processes are important regulators of caste determination and behavioral development in bee colonies (74, 75).

In our study, a total of 28 enrichment pathways were found to be associated with metabolic pathways (Figure 6). They are mainly carbohydrate and lipid metabolism pathways, which account for energy synthesis, release, and storage in living organisms (59, 73). Among them, glycolytic and lipid metabolic pathways are predominantly downregulated. The downregulation of these pathways may imply a decrease in carbohydrate supply and a decrease in energy supply. Since the honey bee abdomen is an important site for breaking down food for energy storage and supply, a negative impact on metabolic pathways could mean a negative effect on the whole organism, which induce development delay in bees. In addition, the expression of genes involved in the metabolism of sulfur compounds were significantly upregulated, which is consistent with the upregulation of the glutathione metabolic pathway, which plays a role not only in metabolism but also in detoxification of reactive oxygen species. Thus, the need for glutathione may be responsible for the accelerated metabolism of sulfur compounds (73).

## Conclusion

Overall, our study showed that exposure of honey bee larvae to high concentrations (1.0 mg/L, T1.0) of thiacloprid significantly delayed development; reduced survival rate, pupation rate and emergence rate, and decreased the body weight and size of honey bees under experimental conditions. Furthermore, we assessed the effects of thiacloprid exposure on changes in gene expression in honey bees using RNA-Seq techniques. We found that thiacloprid exposure impaired honey bee growth and development, metabolism, and immunity by reducing energy supply though the down-regulation of carbohydrate and lipid metabolic pathways, affecting the development of visual and olfactory systems, and activating immune-related signal transduction pathways. In conclusion, these findings could provide support to explore the detailed effects of thiacloprid on honey bee growth and development and immunologic mechanisms. It can be shown that pesticide abuse is closely related to bee colony health, and it also provides a reference for environmental safety assessment in future practical production.

## Data Availability Statement

The datasets presented in this study can be found in online repositories. The names of the repository/repositories and accession number(s) can be found below: https://dataview.ncbi.nlm.nih.gov/object/PRJNA793424?reviewer=ciabkae4vn6999tu1jdkh2544i.

## Author Contributions

Y-JL designed the research. BL, LK, and A-RL performed the research. BL analyzed data. BL and Y-JL wrote the article. Y-JL, QW, and Q-YD reviewed the manuscript. All authors read and approved the final version of the manuscript.

## Funding

This work was supported by National Natural Science Foundation of China (31772683), Chinese Academy of Agricultural Sciences (Elite Youth Program to Y-JL).

## Conflict of Interest

The authors declare that the research was conducted in the absence of any commercial or financial relationships that could be construed as a potential conflict of interest.

## Publisher's Note

All claims expressed in this article are solely those of the authors and do not necessarily represent those of their affiliated organizations, or those of the publisher, the editors and the reviewers. Any product that may be evaluated in this article, or claim that may be made by its manufacturer, is not guaranteed or endorsed by the publisher.

## Supplementary Material

Supplementary Figure 1 Images of honey bee larvae development under different concentrations (0, 0.5 and 1.0 mg/L) of thiacloprid treatments.

Supplementary Figure 2 Images of honey bee pupae body development under three different concentration of thiacloprid treatments.

Supplementary Figure 3 **(A)** Principal component analysis (PCA) of the transcriptomes from different treatment groups. PC1 and PC2 are the top two dimensions of gene differences in these samples, which account for 24.57% and 16.95% of expressed genes, respectively. The number in parentheses indicates the proportion of variance explained by the principal component. **(B)** Venn diagram of up-regulated DEGs and down-regulated DEGs of *A. mellifera* treated with 0.5 mg/L (T0.5) or 1.0 mg/L (T1.0) thiacloprid compared with non-treated control (T0). **(C)** Gene ontology (GO) enrichment classification of DEGs identified in the comparison between treatment groups (T0.5 and T1.0) and control group (T0). There are mainly three categories: molecular function (blue), cellular component (red), and biological process (green). The X-axis indicates the second category of GO terms and the Y-axis indicates the number of DEGs.

Supplementary Table 1 Statistical analysis of transcriptome sequencing data.

Supplementary Table 2 Up- and down-regulation and annotation information of DEGs in different group.

Supplementary Table 3 GO classification of the enriched thiacloprid-responding genes.

Supplementary Table 4 GO classification of the enriched DEGs associated with up- and down-regulation.

Supplementary Table 5 The most enriched KEGG pathway for up- or down-regulated DEGs.

Supplementary Table 6 Sheet1. The most enriched KEGG metabolism-related pathways for up- or down-regulated DEGs. Sheet2. The most enriched KEGG immune-related pathways for up- or down-regulated DEGs. Sheet3. The most enriched KEGG signal transduction related pathways for up- or down-regulated DEGs.
