# Supplementary figures and images for: Exposure of Larvae to Sublethal Thiacloprid Delays Bee Development and Affects Transcriptional Responses of Newly Emerged Honey Bees
Source: Front Insect Sci. 2022 Apr 5;2:844957. doi: 10.3389/finsc.2022.844957 (PMC10926468; doi:10.3389/finsc.2022.844957)

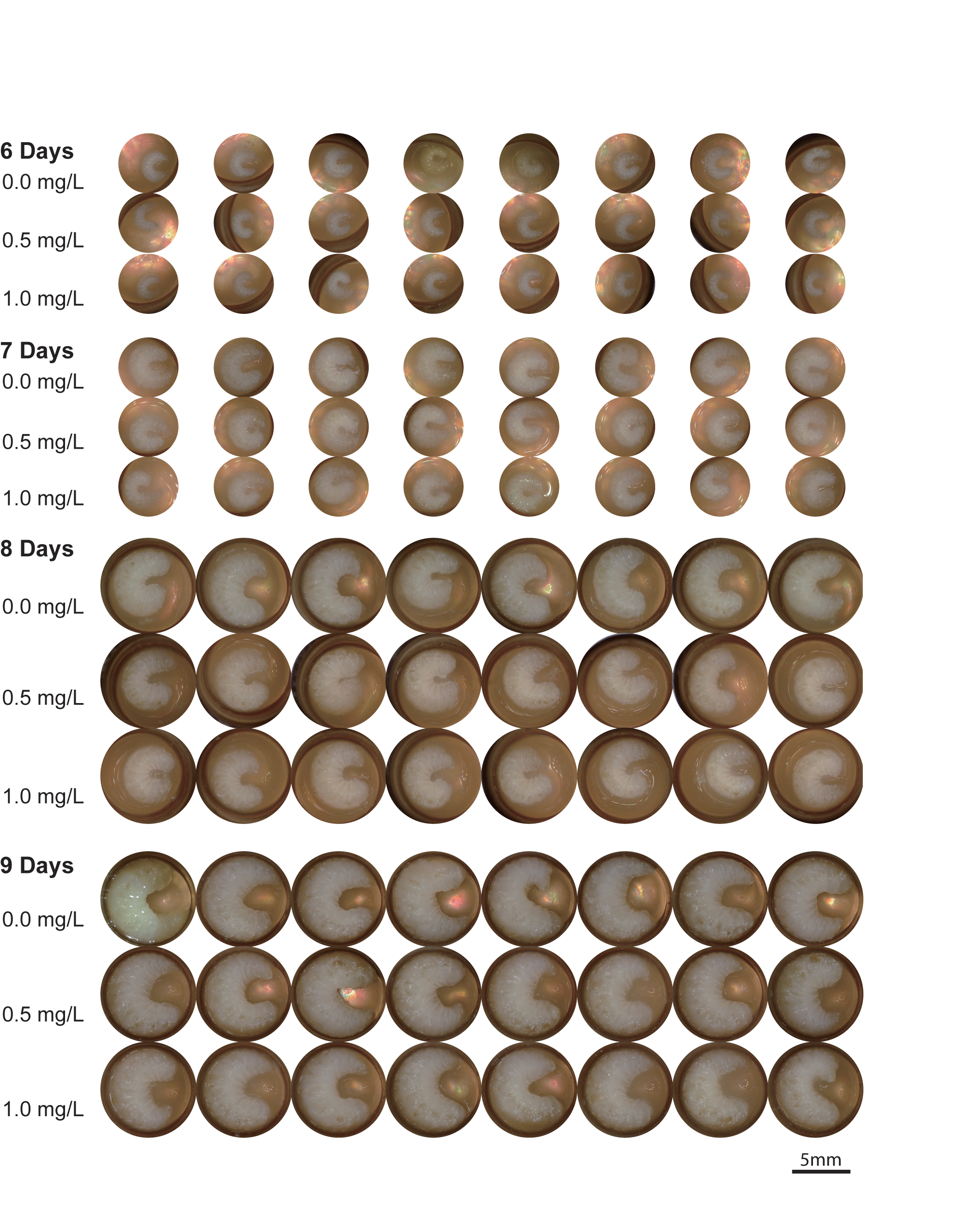

Supplement: Supplementary Figure 1 — Images of honey bee larvae development under different concentrations (0, 0.5 and 1.0 mg/L) of thiacloprid treatments. [file Image_1.TIF]

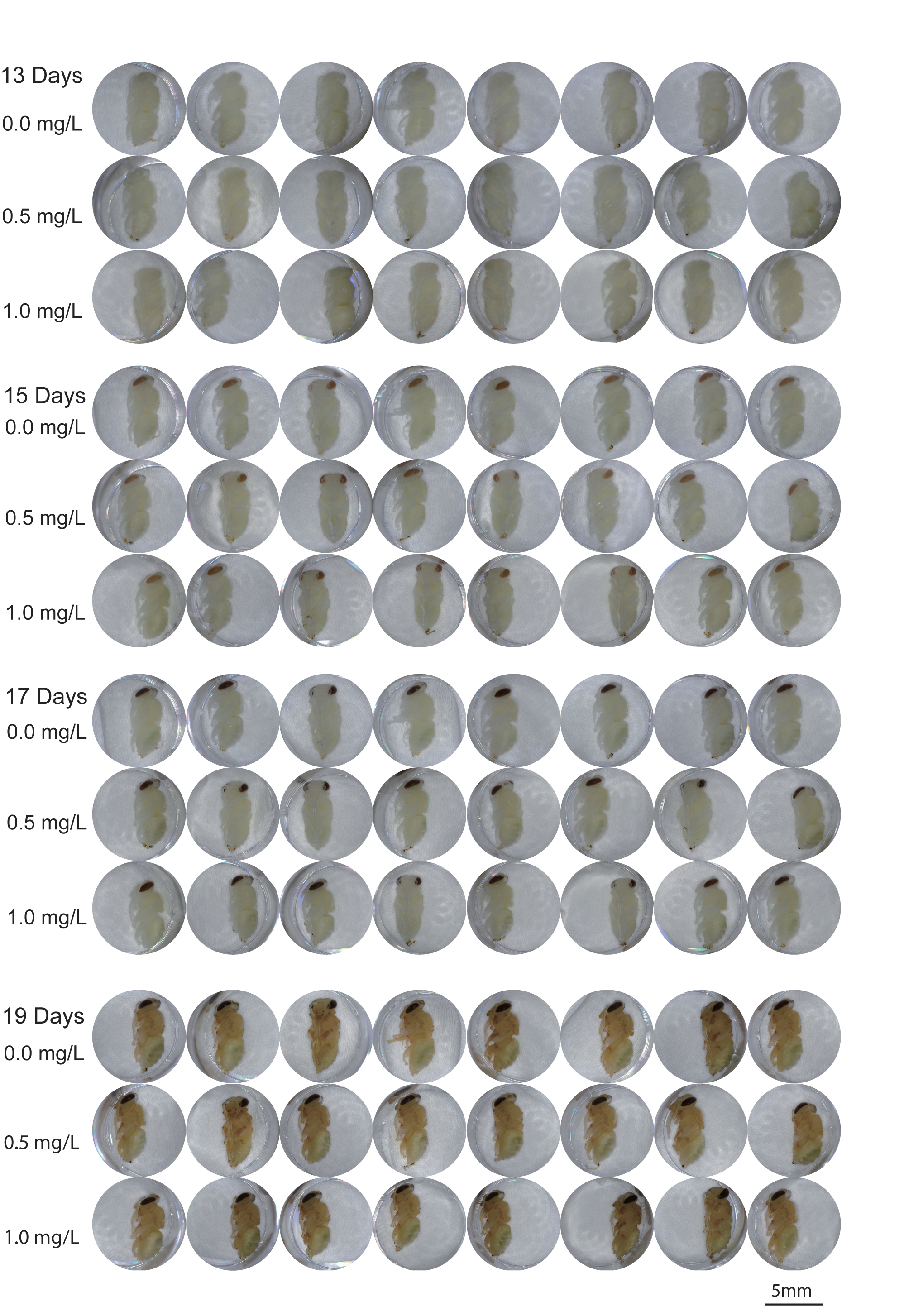

Supplement: Supplementary Figure 2 — Images of honey bee pupae body development under three different concentration of thiacloprid treatments. [file Image_2.TIF]

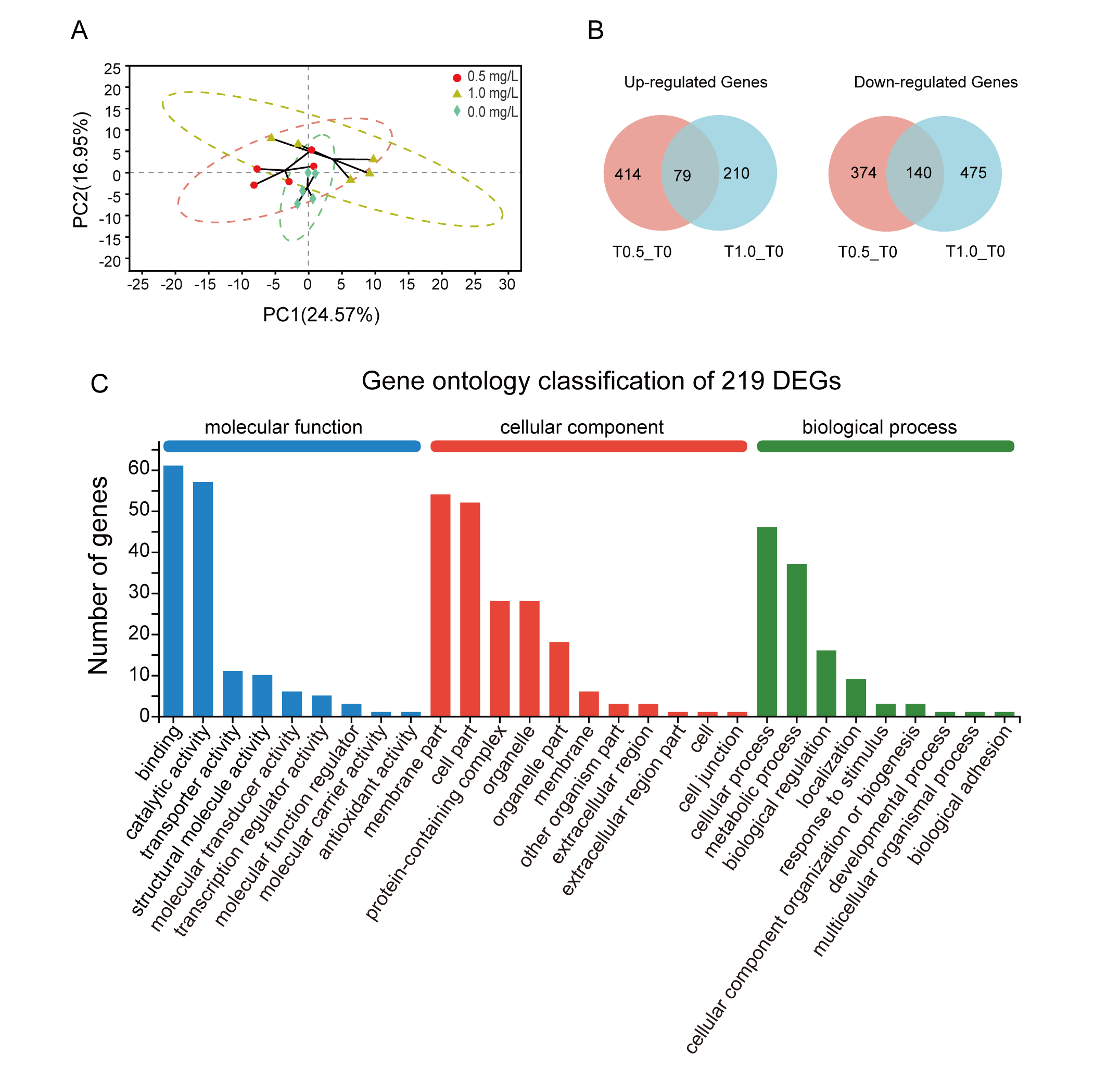

Supplement: Supplementary Figure 3 — (A) Principal component analysis (PCA) of the transcriptomes from different treatment groups. PC1 and PC2 are the top two dimensions of gene differences in these samples, which account for 24.57% and 16.95% of expressed genes, respectively. The number in parentheses indicates the proportion of variance explained by the principal component. (B) Venn diagram of up-regulated DEGs and down-regulated DEGs of A. mellifera treated with 0.5 mg/L (T0.5) or 1.0 mg/L (T1.0) thiacloprid compared with non-treated control (T0). (C) Gene ontology (GO) enrichment classification of DEGs identified in the comparison between treatment groups (T0.5 and T1.0) and control group (T0). There are mainly three categories: molecular function (blue), cellular component (red), and biological process (green). The X-axis indicates the second category of GO terms and the Y-axis indicates the number of DEGs. [file Image_3.TIF]
